# On the Alternation of Generations; or the Propagation and Development of Animals through Alternate Generations, &c.

**Published:** 1846-07

**Authors:** 


On the Alternation of Generations; or, the Propagation and Development of Animals through Alternate Generations: a peculiar Form of fostering the Young in the Lower Classes of Animals. By *Joh. Japetus Sm. Steenstrup*, Lecturer in the Academy of Söro. Translated from the German Version of *C. H. Lorenzen* by *George Busk.* London: printed for the Ray Society. 1845.

This work is calculated to excite much interesting speculation, as exhibiting some new phases in the process of generation, and also as throwing some light upon one of the most obscure points of physiology, the reproductive process of Entozoa. The title, extended as it is, will hardly convey to those unacquainted with the views of the author any very clear notion of the peculiar mode of propagation here described. This cannot, however, be a matter of surprise; for although, as Mr. Busk states, some glimpses of the subject were afforded by the naturalist Chamisso, in his observations on the apparent alternations of generation, so far back as 1819, and by the subsequent researches of Sars and Siebold on the development of the Medusæ, and by those of Lovén on the development of Campanularia, &c., still no one, until the author of the present work, "appears to have entered upon the general question of the existence of an analogous course of development *throughout* the lower classes of animals." —(*Translator's Preface*, p. vi.) As the phenomena described by M. Steenstrup are so novel, we shall probably succeed best in conveying a knowledge of them to our readers, by prefixing a few general remarks illustrative of the relations between them and the ordinary mode of generation.

In all the forms of reproduction with which we are best acquainted, it is seen that the production of the new being is partly effected by powers inherent in the parents, male and female, and partly by powers inherent in the germ or embryo itself; the former effecting the primitive formation of the germ, whether that be accomplished by the male, by the female, or by these conjointly, for all these theories have their advocates; the latter, accomplishing the development or growth of the germ.

It is necessary to bear in mind that the individual modes of generation differ from each other essentially, only by the manner in which this second part of the process (development) is accomplished: it may be oviparous, ovo-viviparous, or viviparous, but in each case the germ is the sole agent of its own growth, however much the mode of its nutrition may vary. This law does not, however, exclude the indirect agency of the parents, principally the mother, in providing some of the necessary conditions—such as a supply of nutritious matter, of heat, of protection, &c. Up to the present time the best known exceptions to these incidental processes being accomplished by the parents, and again we may observe almost exclusively by the mother, are furnished by bees, wasps, ants, and termites, among which interesting animals the curious provision of *nursing* is displayed, that is to say, individuals are met with having no other office than to provide food, heat, and protection for the young; an economy with which all are familiar, who know the proceedings of the bee-hive or ant-hill.

Although, as will afterwards be apparent, this system of nurses is a branch of the propagation of animals by the principle of "alternate generations," we would more specially fix the attention of the reader on the mode of reproduction which takes place among Aphides, because this in reality is a perfect type of the remarkable process elucidated in the present work.

"The propagation of these creatures through a series of generations has been already long known. In the spring, for instance, a generation is produced from the ova, which grows and is metamorphosed, and without previous fertilization, gives birth to a new generation, and this again to a third, and so on, for ten or twelve weeks; so that, in certain species, even as many as nine such preliminary generations will have been observed; but, at last, there always occurs a generation consisting of males and females, the former of which, after their metamorphosis, are usually winged; fertilization and the depositing of eggs takes place, and the long series of generations re-commences in the next year, and in the same order." P. 108.

In order to form a true notion of this most remarkable form of propagation, it is necessary to state that, as no immature or imperfect animal can form germs, the germs of all these generations must have been produced by the perfect and fertile individuals, the several intervening generations, excepting the last, standing simply in the relation of nurses, affording, like the nurse-bees, but *in the interior of their bodies*, nutritious matter, heat, and protection; so that we have here the curious spectacle of animals giving birth to young without being parents, and of young proceeding from individuals of which they are not the offspring.

If this be the true view of the generation of Aphides, it becomes an interesting, and, in connexion with the general laws of ovology, an important question to determine the true sexual nature of the animals forming the intervening generations, and the nature of their retentive genital organs. The history of social bees and wasps shows that the feeders or nurses are females, whose sexual organs remain in an undeveloped state; and Steenstrup affirms, what from this fact we should necessarily infer, that what he well terms "foster-parents," are among the Aphides also imperfect females; indeed, in every similar case, the multitude of individuals, to whom is committed the perfecting of a later generation, "consist invariably of females, the males being apparently excluded from any participation in the office, on which account the males of all the animals among which the system of 'nursing' or of 'feeding' obtains, constitute a very subordinate number." (P. 111.) This conclusion is greatly strengthened by what next follows.

Among the many interesting facts and generalizations of this philosophic investigator, there are none evincing more acumen than those relating to the nature of the retentive organ. M. Steenstrup well observes—

"That the '*nursing*' should be committed to females alone appears to us very natural, since we are acquainted with an organ in them whose natural function would be to perform that office. The generative organs are, indeed, in perfect (female) individuals, divided as it were into two parts of very distinct natures; the *ovarium* for the preparation of the germ and the production of the egg, and the *oviduct* and *uterus* in which the ova are as it were incubated, and the germ and embryo sufficiently developed to allow of its being born. Now, it is actually the case, that no true *ovary* has been discovered in the '*nursing*' generations; on the contrary, the germs, as soon as they are perceptible, are situated in organs which must be regarded as *oviducts* and *uteri*, as, for instance, in the most perfect '*nurses*' we are acquainted with, the *Aphides*." P. 112.

This is a most valuable and distinguishing fact, and not only throws a clear light upon the whole system of alternating generations, but also reconciles with the law already noticed, that imperfect individuals, namely, cannot form germs, what had hitherto appeared to be the anomaly of imperfect Aphides re-producing. We shall have occasion again to notice this point.

It is thus seen that, in this propagation by the agency of nursing animals, the business of generation, so far as the process of developing the new being is concerned, instead of being, as in ordinary re-production, accomplished by one individual or mother, (we exclude for the moment those few cases in which the male assists in nourishing the embryo,) is, in this most remarkable process, divided among several different individuals.

We now proceed more particularly to point out the views of the author, and to detail some of the principal facts that have led to the theory he so ably advocates.

"The special subject of this Essay," observes M. Steenstrup, "is the fundamental idea expressed by the word '*Alternation of Generations*,' or the remarkable, and till now inexplicable natural phenomenon of an animal producing an offspring, which at no time resembles its parent, but which, on the other hand, itself brings forth a progeny, which returns in its form and nature to the parent animal, so that the maternal animal does not meet with its resemblance in its own brood, but in its descendants of the second, third, or fourth degree or generation; and this always takes place in the different animals which exhibit the phenomenon, in a *determinate* generation, or with the intervention of a *determinate* number of generations. This remarkable *precedence* of one or more generations, whose function it is, as it were, to prepare the way for the later, succeeding generation of animals destined to attain a higher degree of perfection, and which are developed into the form of the mother, and propagate the species by means of ova, can, I believe, be demonstrated in not a few instances in the animal kingdom." P. 2.

His investigations concern more especially the phenomena which have been observed in the propagation and development of the Medusæ and of the Claviform Polypes among the Acrita, of the Salpæ among the Mollusca, and of the Trematoda among the Entozoa.

The mode of generation in the Medusæ has for a long time been an enigma among physiologists, and although much of this obscurity was cleared up by the admirable researches of the Norwegian naturalist Sars, and subsequently by those of Siebold, yet it was reserved for the author to trace all the links which connect the ovum with the fully-formed animal. The eggs, after experiencing a certain degree of development first of all in the ovaries, and subsequently in the marsupial vesicles belonging to the oral arms or tentacles, disclose, and bring forth young, not, as might be expected, having the characters of the parent Medusa, but creatures of an oval form, and outwardly resembling the Ciliated Infusoria, such as *Leucophrys* or *Bursaria*. This apparent, but not real, infusorial animalcule soon attaches itself to some fixed object, and by degrees becomes transformed into an animal, having a mouth provided with a number of tentacula amounting ultimately to thirty; in this stage it very much resembles a polype. By a kind of transverse fission this polypiform animal is gradually separated into many portions, which, acquiring independent vitality, are finally separated from each other, and become free swimming animals. Now it is within the abdomen (if such a term may be here applied) of these polypiform Medusæ that the true larvæ of the perfect animals are found; and we must, as far as we at present know, assume that the function of these polypiform individuals is fulfilled when the larvæ have reached a definite development, and that their whole existence has for its object the perfecting of a series of beings of the same species, to which they stand in the relation in what, for want of a better term, may be called "*foster-parents*." (P. 24.)

M. Steenstrup concludes his observations on the propagation of the Medusæ by stating that—

"Since, according to the observations of both Sars and Siebold, only animals which have the power of affixing themselves proceed from the ova of Medusæ, all of which become in the way so often described, polypiform '*nurses*,' which nourish the Medusæ-larvæ from their bodies, a considerable anatomical and physiological difference clearly exists between them, which are *all* of one sex, and the perfect *Medusæ* which are of two sexes. Here, however, we observe, a great natural harmony; since wherever we find the fostering of a brood or fry to be committed to special individuals, these are always of *one* sex, and indeed females in whom the sexual organs of germination remain in an undeveloped state, whilst in consequence of this abortion in the development of these parts, the instinct or *nisus* serving for the preservation of the species takes a peculiar direction." P. 24.

This mode of development has only been seen in two of the most common species of Medusæ (*M. aurita* and *Cyanæa capillata*), but, in all probability, it occurs throughout the whole family. It is further proper to remark that, although the author admits with Sars that the "*nursing*" individuals increase by gemmation, every part of the body being capable of throwing out gemmæ or buds, yet he contends that this fact does not affect the truth of his representation; for the question arises—May not an ovum occasionally contain several embryos, one of which becomes developed at the expense of the others, which make good their individuality at a later period?—*(L. c.)* We are not, it is true, in a position at present to solve this question; but it must be apparent to those who are acquainted with the obscurity overhanging the reproductive process among the most simple animals, some of which are believed to establish the doctrine of equivocal generation, whilst others are pronounced at one time to be fissiparous, at another gemmiparous, and at another oviparous, that these researches of Steenstrup, if ultimately established, must render a review of the whole theory of generation indispensably necessary.

The next class investigated, referring to some families of the multitudinous section of *polypes*, throws further light upon this interesting question. We shall select for illustration one of the bell-shaped polypes, *Campanularia geniculata.* On the branched stem of one of these individuals, are placed a number of cells or cups, shaped like inverted cones or bells, which have a determinate position, and are of three kinds, constituting, according to the author, so many distinct generations. Those of the first order occupy the summits of the branches, and elicit afterwards the larger individuals forming the second class, which, like the bud in a plant, are situated in the axillæ of the branches; in the interior of these axillary polypes are placed the individuals of the third kind or generation, which are in fact the perfect, that is, the reproductive females. The mode in which this curious progeny is produced is, according to Steenstrup, as follows:—between the outer and inner membranes of the second order (axillary polypes) and upon the so-called intestinal tube, small vesicular elevations are formed, each of which becomes included in a transparent membrane or sacculus; within this sac, and in connexion with the vesicular elevation, two perfectly spherical bodies appear; subsequently, the sacculus with its contents approaches the operculum of the axillary cell or polype, and breaks through it, without, however, losing its connexion with the central tube; finally, the apparent sacculus opens, a circlet of about twelve irregularly-toothed tentacula projects around the opening, and it is now seen that the sacculus is in reality a distinct individual. Several such individuals are found within a single axillary polype; and we may now state that they are the completely developed females above noticed, the two spherical bodies being prolific ova, presenting opaque granules (yelk-globules?) and, which is more interesting, the germinal vesicle of Purkinjé. The subsequent steps of the process require to be carefully attended to:—the ova are developed within the parent-body, and when the embryo is matured, it ruptures the delicate membrane of the egg, remains for some time within the parent animal, and then passing out, it enters the water, freely swims about for a time, being provided with vibratile cilia, afterwards attaches itself to some fixed object, and becomes the origin of a new compound polype, on which are formed the three cup-like cells already described. Thus, according to the views here advocated, three distinct and successive generations are engaged in perfecting the fully-developed and fertile female, and are so many sets of nurses or foster-parents.

"Upon reviewing this sketch, it can hardly be considered to present an instance of metamorphosis, since it is not the same *individuals* which in the course of time exhibit various forms, but we see here also, a series of *generations* whose succession in a definite order is necessary to the complete development of the species. The ciliated embryos which, by their adhesion lay the foundation of the polypes, originate only from ova, which are developed in the females. These latter are generated between the inner and outer tunic of the polype in the axillary cells, which polypes may consequently be considered as the '*nurses*' or foster-parents of the female individuals, whilst they are themselves, on the contrary, derived from a previous generation of polypes of a different form, which occupy the extremities of the branches, are of the same shape as the polype of the stem, and must be regarded as of the same kind, although they appear to arise from it by what is called *gemmation.*" P. 37.

In the preceding instances, the theory of Alternate Generations has been traced only among the Radiate sub-kingdom, and consequently in reference to the simplest animal forms. In the Third Chapter, however, comprising an account of the Alternate Generation of the Salpæ, the author proceeds to a more important division, that of the Mollusca. It may be as well to premise that the Salpæ belong to that order of the acephalous molluscous animals, called *Acephala nuda,* and which are divided by Cuvier into two families—the solitary and the aggregated—according as the individuals are met with in an isolated or aggregate condition. We may further remark that there are some peculiarities connected with the mode of generation among these naked Acephala, which have for a long time occupied the attention of Naturalists, and have given rise to many conflicting opinions. We will, in the first place, state the facts that have been ascertained, and then give M. Steenstrup's interpretation of them.

It is well known, as regards the Salpæ, that although they are classed by Cuvier in the solitary division, yet they are also met with joined together, forming long chains consisting of from 20 to 40 individuals or more, possessing organs for attachment; these *salpa-chains* are found in the Mediterranean and warmer parts of the ocean, and all the individuals forming them move with great uniformity, so that the whole chain progresses in a serpentine manner below the smooth and tranquil surface of the water. The most interesting facts hitherto known respecting these curious mollusks were observed by Chamisso. That excellent naturalist ascertained that the solitary salpæ, though never themselves forming parts of a chain, yet always contain a progeny resembling those which do, and, further, that each separate embryo of this progeny is linked together like the individuals forming the salpa-chains; whilst in the associated Salpæ, on the contrary, he found young resembling the solitary ones. "Now, as examination of the free swimming individuals, which presented marks of having been disjointed from a chain, proved that these also contained only solitary pedunculated embryos, Chamisso concluded from his observations, *that all solitary Salpæ, produced associated ones or chains; and, on the contrary, that all the associated Salpæ were parents of solitary ones, and these again of the associated,* and so on." (P. 39.)

This theory of the alternately solitary and associated generations of the Salpæ was so opposed to all then known of the reproductive process, that it is not a matter of surprise that it encountered much opposition. Professor Eschricht, of Copenhagen, although one of Chamisso's opponents, admitted his facts, and added one strongly confirmatory of the truth of his deductions, namely, that in the case of the *Salpa Cordiformis,* "each embryo constituting the embryo-chain, while still enclosed in the body of the mother, presented distinct traces of younger solitary embryos." It is not our object to enter into any of the details of this controversy; we will therefore only mention, as showing the fallacy of Professor Eschricht's arguments, that he supposed the Salpæ, whilst in the associated form, and therefore certainly in an immature condition, might give birth to solitary individuals, and subsequently, becoming detached from each other, that they produced a later generation of associated embryos; a supposition which, as Steenstrup justly remarks, is opposed to the fundamental physiological principle, that no animal is able to propagate before it has attained its perfect form and development.

Such, then, being the fact, for we assume as proved the position of Chamisso, that all Salpa-chains are produced from solitary Salpæ, and all solitary Salpæ from the associated individuals, we come to the author's interpretation. After pointing to the resemblance between the phenomena just described and those noticed in the propagation of the Medusæ, and which assuredly cannot escape observation, M. Steenstrup proceeds—

"I have consequently no hesitation in expressing my belief, that the alternate generation of the *Salpæ,* from solitary and associated individuals, is to be explained in the same way as that of the *Medusæ,* and that the first generation of these animals serves as a series of foster-parents, whose object is, by their furthering the development of the second generation, to conduce to the perfection of the species. I am, however, prepared to admit, that this kind of explanation does not enlarge our knowledge with regard to the propagation of the *Salpæ;* I only think, that the phenomena already known, will have a clearer light thrown upon them from the analogy. Some doubt even must still remain, on the circumstance whether the solitary or the associated *Salpæ* are the foster-parents. In my opinion, however, it is the solitary form rather than the other which should be assumed to be that of the '*nurses.*'" P. 46.

In this last conclusion we cannot agree with the sagacious author, inasmuch as, according to his own remark, the free moving animal must be regarded as more perfect and developed than those which are attached together, and, consequently, as being the true prolific individuals or parents.

We have now to notice one of the most interesting parts of the whole inquiry, the development of the Trematode Entozoa, and especially of the *fluke* or *Liverworm* (Distoma Hepaticum.) Although some links are still wanting, sufficient proofs are adduced to show that the system of "nurses" obtains here, as in so many other of the lower classes of animals. By a very minute and comprehensive investigation, the author appears to have ascertained that several of the Trematoda when young are not connected with any organ, but enjoy free locomotion in water, externally to the animal, which in their future state they infest—(he particularly has examined the flukes of water-snails, such as *Limnæus stagnalis, Planorbis cornea,* &c.)

"In this condition they are provided with a locomotive organ, usually a tail of moderate length, by the waving movement of which the creature propels itself through the water, like a tadpole, to which in its external form it is not dissimilar, except that it is much smaller, and almost microscopic. In this larval state, the *Trematoda* are known to naturalists under the generic name of *Cercaria.* It was well known that this form was not a permanent one, but we were ignorant of the changes which it underwent. As I have been so fortunate as to trace these changes, I will now detail them." P. 53.

This, then, is the first connecting link in the investigation, namely, that *Cercariæ* are in reality the true larvæ of the Distomata infesting water-snails; but they undergo a curious metamorphosis before attaining their ultimate and mature character. On examining with a sufficient magnifying power a portion of the skin of the snail, to which the Cercariæ adhere by their abdominal sucker, it is seen that they make forcible lashing motions of the tail, which organ at length is thus cast off as a lifeless mass, when " the tailless animal itself assumes so completely the appearance of a *Distoma* or *fluke*, that it could not fail of being recognised as belonging to that genus, in case it were met with in this condition in the viscera of other animals." Subsequently the animal, burying itself in the soft integument of the snail, becomes covered with mucus, (which gradually hardens and becomes a nearly transparent case); it is now in fact a pupa, and remains in that state for a period of from two to nine months, when, quitting its puparium, it becomes a true entozoon, the Distoma Hepaticum. We have here the solution of the enigma which puzzled the celebrated helminthologist, Siebold, who could not ascertain what became of the pupæ of the Cercariæ.

These points having been determined, it is necessary next to state that the Cercariæ, although they are thus proved to become Distomata, are not themselves immediately derived from those trematode animals, but from certain parasitic worms found in snails, and described by Bojanus under the somewhat barbarous name of " *King's-yellow-worms*," and which are therefore, in the language of Steenstrup, " nurses," (*Altrices*). This derivation would seem sufficiently complex, but the author is satisfied that even these nurses do not proceed from the ova of the fluke, but from an interposed and pre-existing brood (termed *parent-nurses, Abaltrices*), in the interior of whose bodies he plainly distinguished " a progeny consisting of actual nurses in all stages of development."—(P. 69.) The precise origin of these parent-nurses has not been determined, although it is said there is no doubt they are the immediate offspring of the parent Distoma, being derived in the ordinary mode from ova. In the following table we have endeavoured to place this strange genealogy in a clearer point of view.

1. Distoma Hepaticum—perfect animals, androgynous, reproductive.
2. Parent-nurses (Abaltrices) derived from ova } Imperfect
3. Nurses (Altrices), King's-yellow-worms } females.
4. Cercariæ, true larvæ of fluke.

The author does not describe with any minuteness one of the most important points in the whole theory, the true nature, namely, of the germ from which each generation springs. He says, " at first the germs, from which the Cercariæ are developed, are nearly spherical, and appear to be formed of numerous vesicular globules or cells, which are pretty clearly seen to be all surrounded by a very delicate common membrane: these spherical germs afterwards increase in length, as they are formed into embryos"—(P. 64) ; and a similar obscure account is given of the germs of the "Altrices." The general laws of development, as well as the fact, already stated, that in the similar process among the Campanulate polypes the vesicula Purkinji was detected, lead to the conclusion that each generation springs from true germinal vesicles; and, as imperfect animals are never fertile, these vesicles must all have been derived from the parent distoma; but how the germs of the successive broods are placed as regards each other, we have at present no means of determining. In all cases, however, it is certain that the embryos are lodged when they first appear in the posterior part of the body between the two lateral processes existing there; "it cannot be doubted," adds Steenstrup, "that the germs are always collected in that situation as in a distinct organ, (uterus ?)" As to the mode of birth, it would appear that the Cercariæ quit their "nurses" at a particular depression under what is called the collar, where two apertures are supposed to be placed. An examination of the plates (more particularly Tab. 2, fig. 3, a——h, fig. 5, a——h) confirms the conclusion that, however much the process may deviate from ordinary generation, there is no departure from the fundamental laws.

It has always been a difficult point to comprehend how the Entozoa, which have their habitat in the very substance of organs, the *Trichina spiralis* for example in muscle, effect their entrance. Although this general question cannot be resolved by the proceedings of the Cercariæ infesting aquatic animals, still the mode in which those creatures bury themselves in the skin of the snail on assuming the pupa state, enable us at all events to understand, as the author remarks, how they can enter the nobler internal organs. In this instance it is probable that the instrument for effecting the passage, consists of the circlet of spines on the collar around the oral orifice, since these are found on the Cercariæ, even after they have escaped from the pupa-case, but are cast off after the Distoma has gained its nidus. It is further interesting to remark that, as the Cercariæ of water-snails are proved to undergo their metamorphosis in the interior of the abdominal cavity as well as on the outer surface, there is no difficulty in perceiving that, in higher animals, as mammalia, altrices, or their progeny of larvæ, might be introduced into the alimentary canal, and thence, to borrow a common expression, work their way into the parenchyma of the organs.

The observations of M. Steenstrup respecting the generation and development of Entozoa in general, are of such deep interest that no apology is required for laying them before our readers.

"When a metamorphosis occurs so extensively, or is even universal in one division of the entozoa, the question naturally arises, whether, with respect to the other divisions of that class, it is probable that a similar transformation is effected by a single metamorphosis, or whether even they probably do not attain their development through a series of alternate generations, and exist at first externally to the organisms with which their life is afterwards inseparably connected. Although I cannot commit myself to a precise reply to this query, which is rather beside the especial object of these pages, yet I cannot help giving a few hints on the subject. The *Nematoidea,* which in their adult state often pass from one individual to another, also probably penetrate from without and as embryos, the organism they infest; they do not appear to undergo a true metamorphosis, but to change their skin: I am also unacquainted with any observation which would justify the supposition, that there is in this group any fostering of the young by precedent generations, unless the genus *Sphærularia,* a parasite of the *Hymenoptera,* which Siebold refers to this division of entozoa, be a '*nurse*'; at least it much resembles one, appears to be nearly powerless to perform spontaneous movements, and contains a numerous smooth-skinned progeny, which move about very actively within their parent, but bear no resemblance to it. The *cystic* entozoa on the contrary, betray in many ways that they are a '*nursing*' generation, and especially in the singular circumstance of their being frequently inclosed like boxes one within the other. Probably the full-grown animal of this division is quite unknown, and it is not unlikely, that in course of time, it may happen with them as it has with the whole division of the '*asexual*' Trematoda of Siebold, viz. *Cercaria, Leucochloridium,* &c., that they must be rejected from the system as being earlier forms of development, or earlier generations of other animals. The *Echinorhynchi* present several phenomena which are interesting with regard to our object, viz., the remarkable incubation or breeding which takes place between the skin and the viscera, and the inclusion and incipient development of the ova in the so-called '*loose ovaries,*' during the continued growth of the latter. I must confess, that I look upon these oval bodies much rather as individuals which will never quit the parent animal, than as '*ovaries*;' and till their true nature is known I shall regard them as such, and consequently consider most of the *Echinorhynchi* hitherto known, as '*nurses.*' It is confessedly uncertain whether the *Echinorhynchi* spend part of their life externally to the organism which they inhabit as full-grown animals, or not; it is however very probable they do so, as the embryo attains no real development in the ova so long as these are in the *Echinorhynchus*; and the ova are met with in the mucus of the stomach and in the excrements, by thousands, in the same condition, so that the development of the young in the ova and their escape from them, certainly occurs very long after the ova have reached the water." P. 101.

"Lastly, with respect to the metamorphosis of the *Cestoid* worms, we have an example of it presented to us in Miescher's interesting memoir on the forms which the genus *Tetrarhynchus* passes through; but which also in fact includes nearly all we know about them; whilst the great work of Professor Eschricht, upon the genus *Bothriocephalus* of this family, has given an entirely new view of those animals, constituted of almost innumerable joints, so that they are to be regarded, according to his anatomical investigations, not as single but as *compound* animals, viz., compound *Trematoda* or *flukes,* so that each joint is to be compared with a *distoma,*—a view of their nature to which Baer had some time before alluded, and which had also struck Creplin and Mehlis, on account of the resemblance of the sexual organs, but which is now for the first time entitled to the greatest attention, having been rendered probable by so many anatomical researches. However, I cannot for my part entirely coincide in this view of the *Cestoid* family of worms, for in whatever way the joints and their reciprocal connexion are considered, compound animals are presented, whose construction is entirely different from that of all other animals. The tapeworm is certainly not a single individual, but consists of several; that is, it is constituted of the head, which is an animal, and of the progeny derived from it. This view is much supported, it is even proved by the fact, that the offspring (joints) in a state of progressive development, never actually become animals similar to that from which they spring (the cephalic joint,) which alone remains dissimilar to all the rest, never acquires any developed sexual organs, and consequently never generates any ova, which the others produce in great abundance; whilst the cephalic joint is furnished with a mouth and suctorial acetabula, proceeds from an egg, and is the animal upon which the development of all the rest depends, and is thus in fact one of those animals to which we have in the preceding pages given the name of '*nurse.*' If this view be correct, which time will determine, it will be seen that the *Bothriocephali,* just as all the other animals mentioned in this Essay, which are developed through alternating generations, present quite another and more significant resemblance to plants, than that set up by Professor Eschricht. The individuals which are fostered by, and *appear* to proceed from the head by the so-called transverse division, thus attain such a degree of perfection, that the ova are fully formed in them even before they become detached from the '*nursing animal*'; and, as defensive cases for the ova, they are passed in the natural way from the animal which they have infested, in order probably to reach, eventually, another similar animal, under another form, and as other individuals." P. 104.

In concluding our notice of this remarkable work, we are anxious to point to some of the more important results which it seems to establish. And, first of all, it will not be superfluous to state, as corroborative of the author's views, that many of the facts upon which they repose have been ascertained by other and earlier investigators, and are acknowledged to be well founded; thus, whatever doubts may be entertained, they must have reference not to the phenomena themselves, but to the way in which these are to be interpreted. They, the phenomena, have usually been regarded as instances merely of metamorphosis or transformation; but, as M. Steenstrup justly observes, the essential objection that a *metamorphosis* can only imply changes which occur in the *same* individual, has been overlooked; and, further, that when from an individual other individuals originate, something more than a metamorphosis is concerned.

"Thus, it is quite erroneous to term a *Scyphistoma* the larval condition of *Medusa aurita*, since a *Scyphistoma* never becomes a *medusa*, but is the *quasi* mother of some scores of them. Sars and Lovén have taken a more correct view of the relation in which these creatures stand with regard to each other, seeing in the development of the *Medusæ* and *Campanulariæ* a series of *generations* undergoing metamorphosis. It is of the more essential importance that the distinction between an alternation of generation and a metamorphosis should be understood, because a metamorphosis may readily occur in one or other of the alternating generations themselves, as we see, for example, in the *Distomata* and *Aphides*." P. 6.

In the next place it is to be remarked that, however bizarre and exceptional the phenomena in question may seem to be, they in reality fall within the general laws of development, of the fixity and universality of which, when correctly viewed, they offer a most striking example. It is here, as in all other sciences, perceived, that the admission of exceptions is indicative of imperfect knowledge and of limited observation; for, in proportion as facts multiply and afford the materials for induction, what had hitherto seemed to be isolated and exceptional instances are found to form but a part of a whole series of phenomena, all conformable to fixed and undeviating laws. In the case before us it would have been a real exception, if the imperfect animals, forming the intervening generations, had been prolific; but it has been already shown that they have no ovaries (or more probably that they possess those organs in an undeveloped state), and we may, with the author, further assume, "that the 'nursing' individuals are never themselves gemmiparous, but that they are born with germs in the organs (uteri) in which the embryos are afterwards nourished."

In connexion with this way of viewing the subject, it is not one of the least interesting results that the curious economy of the bee and wasp, of ants and termites, which has up to the present time been regarded as a thing sui generis, is shown to be but a part of a mode of generation extensively prevailing among the lower animals, and which is itself only a modification of, and not a deviation from, the normal female action—the nutrition of the germ.

We have already pointed out, what cannot have escaped the notice of our readers, that many instances of what have hitherto been regarded as gemmiparous and fissiparous generation, will probably be shown to belong to the system of alternate generation. Nor is it attaching too much importance to these researches to affirm, that they will throw considerable light upon some of the still obscure parts of the reproductive process, and, among others, on equivocal generation.

Among what the author properly styles secondary results, are some highly important facts: such as "that the *Cercariæ* are *larvæ* of entozoa, of the genus *Distoma*, and in fact of those species which inhabit the interior of fresh-water snails (in the liver, &c.); *that* the entozoa pass part of their existence in a state of freedom in the water external to the snail, which they afterwards inhabit, and that they re-enter them from without; *that* whole established divisions of families of animals must be abolished, since they include only undeveloped forms, or forms which bear the same relation to the true and perfect form of the species, that the 'workers' among ants and bees bear to the fertile females of those insects; and, finally, *that* several forms which have been considered as of different species and genera are seen to be stages in the development of one and the same animal." To this enumeration must also be added the important light which is thrown by these inquiries into the character of what have been regarded, either as a sexual or hermaphrodite animals, but which, at all events in many instances, are nothing else than imperfectly developed-females.

M. Steenstrup thus philosophically sums up his researches:—

"I conclude with the remark, that, inasmuch as in the system of '*nursing*,' the whole advancement of the welfare of the young is effected only by a still and peaceful organic activity, is only *a function of the vegetative life* of the individual, so also all those forms of animals in whose development the '*nursing*' system obtains, actually remind us of the propagation and vital cycle of plants. For it is peculiar to plants and as it were their special characteristic, that the germ, the primordial individual in the vegetation or seed, is competent to produce individuals which are again capable of producing seeds or individuals of the primary form or that to which the plant owed its origin, only by the intervention of a whole series of generations. It is certainly the great triumph of Morphology, that it is able to show how the plant or tree (that colony of individuals arranged in accordance with a simple vegetative principle, or fundamental law,) unfolds itself through a frequently long succession of generations, into individuals, becoming constantly more and more perfect, until, after the immediately precedent generation, it appears as *Calyx* and *Corolla* with perfect male and female individuals; stamens and pistils (so that even in the vegetable kingdom the grosser hermaphroditism does not obtain, which is still supposed to take place in the animal,) and after, the fructification brings forth seed, which again goes through the same course. It is this great and significant resemblance to the vegetable kingdom, which in my opinion is presented by the *entozoa*, and all '*nurse*' generations, and to which I have alluded in the preceding Essay; I might almost say, that the condition of continued dependance incidental to the animal life, is, to a certain extent, one of less perfection than that which is presented in the progressive elevation in development effected by the agency of the vegetative life." P. 115.

We have extended this article beyond the limits orignally designed for it, having ourselves derived the greatest satisfaction, and we may add instruction, from perusing the deeply scientific and profound work of M. Steenstrup; and we should not do justice to our own sentiments, if we did not congratulate the Ray Society, and that excellent observer Mr. Busk, on having heen the medium of making known to the English public, a series of reseaches equally interesting to the physiologist, the naturalist, and the pathologist.

